# Measurement of blood pressure for the diagnosis and management of hypertension in different ethnic groups: one size fits all

**DOI:** 10.1186/s12872-017-0491-8

**Published:** 2017-02-08

**Authors:** Paramjit Gill, M. Sayeed Haque, Una Martin, Jonathan Mant, Mohammed A. Mohammed, Gurdip Heer, Amanpreet Johal, Ramandeep Kaur, Claire Schwartz, Sally Wood, Sheila M. Greenfield, Richard J. McManus

**Affiliations:** 1 Primary Care Clinical Sciences, University of Birmingham, Edgbaston, Birmingham, B15 2TT UK; 2 Institute of Clinical Sciences, University of Birmingham, Edgbaston, Birmingham, B15 2TT UK; 3 Primary Care Unit, University of Cambridge, Cambridge, CB2 0SR UK; 4 School of Health Studies, University of Bradford, Bradford, BD7 1DP UK; 5 Primary Care Health Sciences, NIHR School for Primary Care Research, University of Oxford, Radcliffe Observatory Quarter, Woodstock Rd, Oxford, OX1 2GG UK

**Keywords:** Diagnosis of hypertension, Ethnic group

## Abstract

**Background:**

Hypertension is a major risk factor for cardiovascular disease and prevalence varies by ethnic group. The diagnosis and management of blood pressure are informed by guidelines largely based on data from white populations. This study addressed whether accuracy of blood pressure measurement in terms of diagnosis of hypertension varies by ethnicity by comparing two measurement modalities (clinic blood pressure and home monitoring) with a reference standard of ambulatory BP monitoring in three ethnic groups.

**Methods:**

Cross-sectional population study (June 2010 - December 2012) with patients (40–75 years) of white British, South Asian and African Caribbean background with and without a previous diagnosis of hypertension recruited from 28 primary care practices. The study compared the test performance of clinic BP (using various protocols) and home-monitoring (1 week) with a reference standard of mean daytime ambulatory measurements using a threshold of 140/90 mmHg for clinic and 135/85 mmHg for out of office measurement.

**Results:**

A total of 551 participants had complete data of whom 246 were white British, 147 South Asian and 158 African Caribbean. No consistent difference in accuracy of methods of blood pressure measurement was observed between ethnic groups with or without a prior diagnosis of hypertension: for people without hypertension, clinic measurement using three different methodologies had high specificity (75–97%) but variable sensitivity (33–65%) whereas home monitoring had sensitivity of 68–88% and specificity of 64–80%. For people with hypertension, detection of a raised blood pressure using clinic measurements had sensitivities of 34–69% with specificity of 73–92% and home monitoring had sensitivity (81–88%) and specificity (55–65%).

**Conclusions:**

For people without hypertension, ABPM remains the choice for diagnosing hypertension compared to the other modes of BP measurement regardless of ethnicity. Differences in accuracy of home monitoring and clinic monitoring (higher sensitivity of the former; higher specificity of the latter) were also not affected by ethnicity.

**Electronic supplementary material:**

The online version of this article (doi:10.1186/s12872-017-0491-8) contains supplementary material, which is available to authorized users.

## Background

Hypertension is the leading risk factor for cardiovascular disease (CVD), accounting for approximately 45% of global CVD morbidity and mortality [1]. The prevalence of hypertension in various regions of the world has been reported [2, 3] and there are striking differences in both blood pressure (BP) level and hypertension prevalence between ethnic groups. For example, adults of West African descent in Europe and North America, whether they come directly from Africa or indirectly from the Caribbean, generally have higher BP and a higher prevalence of hypertension than those of European descent, with this being seen at all ages in North America and only from adulthood in the UK [4, 5].

The diagnosis and management of BP in the UK are informed by guidelines largely based on research from white populations [6]. These guidelines recommend diagnostic and treatment thresholds for hypertension on the basis of office BP and 24 h Ambulatory blood pressure monitoring (ABPM) or home BP monitoring. The need to adjust between clinic and “out-of-office” thresholds for diagnosis makes this particularly important and current recommendations were derived from Australian data gathered in a population that was 82% white and 15% Asian [7]. They suggest a decrease of 5/5 mmHg when converting from clinic to –out-of-office measured BP at lower levels (stage 1 threshold) and a corresponding decrease of 10/5 mmHg at higher levels (stage 2 threshold). We have shown that BP differences between ethnic groups are small [8] and currently ethnicity is not considered in the specification of these UK thresholds, treatment targets or adjustment factors. Hence this study addressed whether accuracy of diagnosis of hypertension using home monitoring and clinic BP are comparable for White British (WB), South Asians (SA) and Black African Caribbean (AC) UK ethnic groups using ABPM as a reference standard.

## Methods

The design and results of the BP in different ethnic groups (BP-Eth) study has previously been published [8, 9]. This was a cross-sectional population study which took place between June 2010 - December 2012 involving people recruited from 28 general practices in Central England.

### Population

The study population comprised two groups aged between 40 and 75 years: The first group were not known to be hypertensive (NHT) and the second had been previously labelled as hypertensive via a clinical code (HT). Participants were drawn from one of four ethnic groups namely WB, SA, AC and White Irish (WI). WI participants were excluded from this analysis due to insufficient numbers recruited (51). Patients that were unable to give consent to the study, belonged to a different ethnic group or whose general practitioner (GP) felt they were unable to take part were excluded.

### Procedures

The study compared BP monitored in a clinic setting and from home-monitoring with ambulatory measurements. Recruitment was from those responding to a postal survey who indicated a willingness to participate in a validation study [8, 9]. Respondents were purposefully sampled from those willing to take part on the basis of ethnicity and hypertension status and invited to attend clinics run at their own practices by research nurses using standardised protocols.

Following informed consent and a five minute rest, six sets of BP measurements were taken by the research nurse at each of three clinic visits (BpTru Medical Devices BPM-100) [10]. On the first occasion BP was measured simultaneously on both arms and thereafter it was measured on the non dominant arm unless the difference in systolic pressure was >20 mmHg between both arms in which case it was measured in the arm with the higher reading [6].

Participants were fitted with an ambulatory monitor (Spacelabs 90217-1Q) [11] (or given a home monitor (Microlife watch BP home)) [12] on either the first or second visit. The third and final visit took place 10 days after the first to allow adequate time for both ambulatory and home BP measurements to be undertaken. The order of ambulatory and home monitoring was varied so that approximately half of the participants had each method first. All staff involved in the study underwent training by the lead research nurse in order to ensure a consistent approach.

Ambulatory readings were recorded at half hourly intervals during the day (7 am to 11 pm) and hourly overnight and the mean daytime BP calculated. Home measurements were taken twice each morning and evening for 1 week, the first days readings discarded and the mean of the remaining readings calculated. For both home-monitored and day time mean ambulatory blood pressure, standard editing criteria were applied: ABPM readings were considered to be valid if there were 14 or more day-time (7 am to 11 pm) readings for a patient (threshold 135/85 mmHg) [13]. Home-monitored readings, minimum of 12 readings, were considered valid if there were 4 or more days readings using the average excepting the first day’s readings (threshold 135/85 mmHg) [14].

Clinic measurement was defined in three ways: the mean of the 2^nd^ and 3^rd^ reading averaged over the 3 days (Clinic23: standardised clinic, threshold 140/90 mmHg) representing recommended clinic BP measurement for diagnosis; the mean of the 2^nd^ to 6^th^ reading averaged over three occasions (Clinic26: research clinic, threshold 135/85 mmHg) and the first reading taken on the first day (ClinicD1R1: casual clinic, threshold 140/90 mmHg), which was expected to accentuate any white coat effect.

### Outcome measures

The primary outcome was the diagnostic test performance of various measures of clinic and home-monitored BP compared to the reference standard (mean daytime ambulatory BP) considering both diagnosis of hypertension in those not previously diagnosed and identification of poor control in those known to be hypertensive, using a threshold of 140/90 mmHg for clinic readings and 135/85 mmHg for out-of-office measurement [6]. The same measurement methodology and thresholds were used for hypertensive and non-hypertensive individuals.

### Statistical analysis

People with and without a previous diagnosis of hypertension were analysed separately and the impact of ethnicity was assessed.

The continuous response variable was systolic or diastolic BP. The study design involved clustering effects (BP readings nested within days and patients), so we used a hierarchical linear statistical model to reflect the design. A 3-level hierarchical model was developed, with level 1 as the BP readings, level 2 as the day (the readings were taken) and level 3 as the patient. All models had a pre-specified set of covariates: ethnicity, age, sex, marital status, deprivation (IMD 2007), body mass index, smoking status, alcohol consumption, cholesterol, cardiovascular disease, chronic kidney disease, diabetic status, and hypertension status. Five separate models were constructed, one for each method—ABPM, clinic23, clinic26, clinic D1R1 and home monitoring. For clinic D1R1 there was no hierarchical structure as there was a single observation per participant (a simple linear regression model was used). Participants with complete data were included in the analyses. All analyses were undertaken in Stata (release 12) [15, 16].

Sensitivity, specificity, likelihood ratio of a positive test (LR + ve) and likelihood ratio of a negative test (LR –ve) for either a diagnosis of hypertension or confirmation of raised BP in those known to be hypertensive, were calculated for each ethnic group and also for all samples for Clinic23, Clinic26, ClinicD1R1 and home monitoring using mean day-time ABPM as a reference standard.

## Results

### Baseline data: demographics and past medical history

A total of 551 patients had complete records in the study (246 WB, 158 AC, and 147 SA) (Table 1). More hypertensives than non-hypertensives had complete data in each group. The WB group were older than the other two and more likely to drink alcohol. The SA group had a lower prevalence of smoking but were more likely to be diabetic.

**Table 1:** Characteristics of study population

	Not known to be hypertensive				Diagnosed hypertensive				All
	WB	SA	AC	All	WB	SA	AC	All	
n	98	55	58	211	148	92	100	340	551
Age	59.4 (9.4)	53.6 (8.9)	51.7 (8.6)	55.8 (9.7)	64.4 (7.3)	60.0 (8.5)	59.4 (9.0)	61.8 (8.5)	59.5 (9.4)
Male	43 (43.9)	30 (54.6)	26 (44.8)	99 (46.9)	83 (56.1)	56 (60.9)	39 (39.0)	178 (52.4)	277 (50.3)
Married/Cohabiting	68 (69.4)	46 (83.6)	33 (56.9)	147 (69.7)	88 (59.5)	85 (92.4)	30 (30.0)	203 (59.7)	350 (63.5)
Employed or F.T. Student or Housewife/husband	44 (44.9)	43 (78.2)	41 (70.7)	128 (60.7)	34 (23.0)	45 (48.9)	38 (38.0)	117 (34.4)	245 (44.5)
Deprivation	34.7 (15.6)	40.8 (19.6)	50.9 (13.7)	40.7 (17.5)	36.4 (17.7)	41.7 (16.5)	49.1 (15.7)	41.5 (17.6)	41.2 (17.6)
Smoker	15 (15.3)	2 (3.6)	4 (6.9)	21 (10.0)	28 (18.9)	4 (4.4)	17 (17.0)	49 (14.4)	70 (12.7)
Alcohol									
Non-drinker	32 (32.7)	40 (72.7)	34 (58.6)	107 (50.2)	55 (37.2)	68 (73.9)	59 (59.0)	182 (53.5)	288 (52.3)
Mild/Moderate drinker	44 (44.9)	14 (25.5)	20 (34.5)	78 (37.0)	66 (44.6)	19 (20.7)	37 (37.0)	122 (35.9)	200 (36.3)
Heavy drinker	22 (22.5)	1 (1.8)	4 (6.9)	27 (12.8)	27 (18.2)	5 (5.4)	4 (4.0)	36 (10.6)	63 (11.4)
BMI	27.8 (4.3)	27.0 (3.1)	29.7 (6.2)	28.1 (4.7)	30.1 (4.8)	28.9 (3.8)	30.5 (5.3)	29.9 (4.7)	29.2 (4.8)
Normal (19–25)	26 (26.5)	12 (21.8)	8 (13.8)	46 (21.8)	20 (13.5)	14 (15.2)	18 (18.0)	52 (15.3)	98 (17.8)
Overweight	46 (46.9)	36 (65.5)	28 (48.3)	110 (52.1)	58 (39.2)	45 (48.9)	28 (28.0)	131 (38.5)	241 (43.7)
Very overweight	26 (26.5)	7 (12.7)	22 (37.9)	55 (26.1)	70 (47.3)	33 (35.9)	54 (54.0)	157 (46.2)	212 (38.5)
High Cholesterol	14 (14.3)	19 (34.6)	9 (15.5)	42 (19.9)	72 (48.7)	45 (48.9)	28 (28.0)	145 (42.7)	187 (33.9)
Cardiovascular Disease	9 (9.2)	6 (10.9)	3 (5.2)	18 (8.5)	42 (28.4)	16 (17.4)	13 (13.0)	71 (20.9)	89 (16.2)
Diabetes	3 (3.1)	7 (12.7)	2 (3.5)	12 (5.7)	22 (14.9)	34 (37.0)	22 (22.0)	78 (22.9)	90 (16.3)
Chronic Kidney Disease	4 (4.1)	1 (1.8)	6 (10.3)	11 (5.2)	12 (8.1)	4 (4.4)	13 (13.0)	29 (8.5)	40 (7.3)

Numbers are Mean (SD) for continuous variables and Number (Percentage) for categorical variables, Index of Multiple Deprivation 2007 score

*WB* White British, *SA* South Asian, *AC* African Caribbean

The differences seen between ethnic groups overall were largely mirrored within those with and without hypertension, although hypertensives were older, had more co-morbidities and were less likely to be working (Table 1).

As there was no difference between raw and modelled data, modelled data are provided (see Additional file 1 of diagnostic output of raw data at different BP thresholds).

### Diagnostic test performance for raised BP without a diagnosis of hypertension

**Fig. 1:**
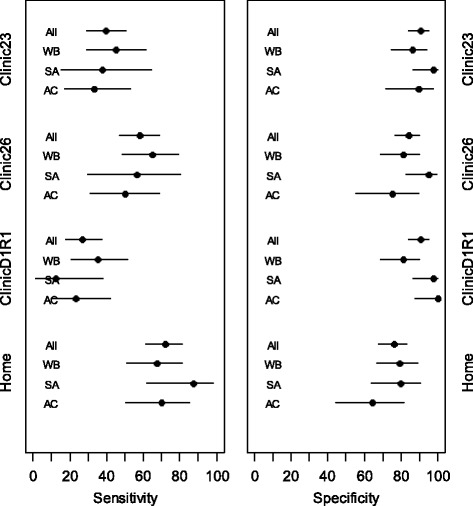
Sensitivity and specificity non-hypertensive

The results for diagnostic test performance compared to an ambulatory BP above 135/85 mmHg in people not known to be hypertensive – i.e. for a diagnosis of hypertension - were similar for each ethnic group within each alternative method of measurement evaluated. Considering the individual measurement methods for all patients combined, there was low sensitivity for clinic23 with high specificity (Fig. 1; Table 2). For clinic26 measurement, the sensitivities were better than those of Clinic23 with relatively lower specificity. For ClinicD1R1 sensitivity was much lower particularly amongst SAs but there were high specificities. For home-monitoring, sensitivity was higher and specificity was lower than clinic measurements.

**Table 2:** Non-Hypertensive: diagnostic performance for hypertension defined by mean daytime ABPM

Ethnicity	Sensitivity (95% CI)	Specificity (95% CI)	LR + ve (95% CI)	LR -ve (95% CI)
a) ABPM v Clinic23 on three occasions (thresholds: ABPM 135/85 mmHg, Clinic23 140/90 mmHg)				
All sample (*n* = 211)	39.5% (29.2–50.7%)	90.4% (83.8–94.9%)	4.12 (2.26–7.49)	0.67 (0.56–0.80)
WB (*n* = 98)	45.0% (29.3–61.5%)	86.2% (74.6–93.9%)	3.26 (1.57–6.76)	0.64 (0.47–0.86)
SA (*n* = 55)	37.5% (15.2–64.6%)	97.4% (86.5–99.9%)	14.63 (1.91–111.97)	0.64 (0.44–0.94)
AC (*n* = 58)	33.3% (17.3–52.8%)	89.3% (71.8–97.7%)	3.11 (0.95–10.15)	0.75 (0.56–0.99)
b) ABPM v Clinic26 on three occasions (thresholds: ABPM 135/85 mmHg, Clinic26 135/85 mmHg)				
All sample (*n* = 211)	58.1% (47.0–68.7%)	84.0% (76.4–89.9%)	3.63 (2.34–5.64)	0.50 (0.38–0.65)
WB (*n* = 98)	65.0% (48.3–79.4%)	81.0% (68.6–90.1%)	3.43 (1.92–6.11)	0.43 (0.28–0.67)
SA (*n* = 55)	56.3% (29.9–80.2%)	94.9% (82.7–99.4%)	10.97 (2.66–45.26)	0.46 (0.26–0.81)
AC (*n* = 58)	50.0% (31.3–68.7%)	75.0% (55.1–89.3%)	2.00 (0.96–4.17)	0.67 (0.44–1.01)
c) ABPM v ClinicD1R1 (thresholds: ABPM 135/85 mmHg, ClinicD1R1 140/90 mmHg)				
All sample (*n* = 211)	26.7% (17.8–37.4%)	90.4% (83.8–94.9%)	2.79 (1.47–5.29)	0.81 (0.70–0.93)
WB (*n* = 98)	35.0% (20.6–51.7%)	81.0% (68.6–90.1%)	1.85 (0.94–3.64)	0.80 (0.62–1.04)
SA (*n* = 55)	12.5% (1.6–38.3%)	97.4% (86.5–99.9%)	4.88 (0.47–50.05)	0.90 (0.74–1.09)
AC (*n* = 58)	23.3% (9.9–42.3%)	100% (87.7–100%)	. (. - .)	0.77 (0.63–0.93)
d) ABPM v Home-monitoring for 1 week (thresholds: ABPM 135/85 mmHg, Home 135/85 mmHg)				
All sample (*n* = 211)	72.1% (61.4–81.2%)	76.0% (67.5–83.2%)	3.00 (2.14–4.21)	0.37 (0.26–0.52)
WB (*n* = 98)	67.5% (50.9–81.4%)	79.3% (66.6–88.8%)	3.26 (1.89–5.64)	0.41 (0.26–0.65)
SA (*n* = 55)	87.5% (61.7–98.4%)	79.5% (63.5–90.7%)	4.27 (2.24–8.13)	0.16 (0.04–0.58)
AC (*n* = 58)	70.0% (50.6–85.3%)	64.3% (44.1–81.4%)	1.96 (1.13–3.40)	0.47 (0.25–0.86)

### Diagnostic test performance for controlled BP in people with a diagnosis of hypertension

**Fig. 2:**
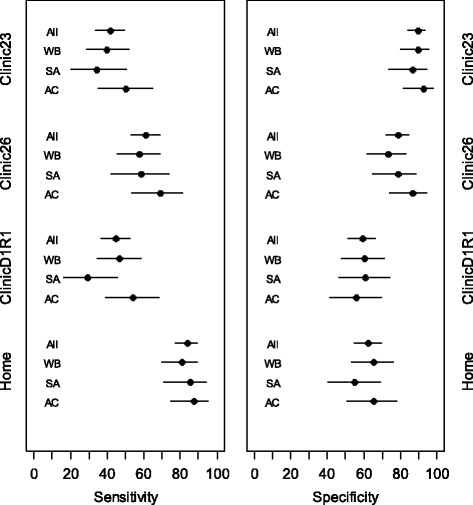
Sensitivity and specificity hypertensive

The results for diagnostic test performance compared to an ambulatory BP above 135/85 mmHg in people known to be hypertensive –i.e. for a confirmation of poor control - were also similar for each ethnic group within each alternative method of measurement evaluated. As with those not known to be hypertensive, overall clinic23 measurement had low sensitivity with high specificity. Clinic26 measurement had moderate sensitivity and lower specificities whereas ClinicD1R1 measurement had similarly low sensitivities and specificities. Home-monitoring had high sensitivities but lower specificities (Fig. 2; Table 3).

**Table 3:** Hypertensive

Ethnicity	Sensitivity (95% CI)	Specificity (95% CI)	LR + ve (95% CI)	LR -ve (95% CI)
a) ABPM v Clinic23 on three occasions (thresholds: ABPM 135/85 mmHg, Clinic23 140/90 mmHg)				
All sample (*n* = 340)	41.4% (33.7–49.4%)	89.3% (83.8–93.4%)	3.87 (2.44–6.16)	0.66 (0.57–0.75)
WB (*n* = 148)	39.7% (28.5–51.9%)	89.3% (80.1–95.3%)	3.72 (1.82–7.60)	0.67 (0.55–0.83)
SA (*n* = 92)	34.1% (20.1–50.6%)	86.3% (73.7–94.3%)	2.49 (1.11–5.59)	0.76 (0.60–0.98)
AC (*n* = 100)	50.0% (35.2–64.8%)	92.3% (81.5–97.9%)	6.50 (2.43–17.37)	0.54 (0.40–0.73)
b) ABPM v Clinic26 on three occasions (thresholds: ABPM 135/85 mmHg, Clinic26 135/85 mmHg)				
All sample (*n* = 340)	61.1% (53.1–68.7%)	78.7% (71.9–84.4%)	2.86 (2.1–3.89)	0.49 (0.40–0.61)
WB (*n* = 148)	57.5% (45.4–69.0%)	73.3% (61.9–82.9%)	2.16 (1.41–3.30)	0.58 (0.43–0.78)
SA (*n* = 92)	58.5% (42.1–73.7%)	78.4% (64.7–88.7%)	2.71 (1.51–4.86)	0.53 (0.36–0.78)
AC (*n* = 100)	68.8% (53.7–81.3%)	86.5% (74.2–94.4%)	5.11 (2.50–10.44)	0.36 (0.23–0.56)
c) ABPM v ClinicD1R1 (thresholds: ABPM 135/85 mmHg, ClinicD1R1 140/90 mmHg)				
All sample (*n* = 340)	44.4% (36.6–52.4%)	59.0% (51.4–66.3%)	1.08 (0.85–1.39)	0.94 (0.78–1.13)
WB (*n* = 148)	46.6% (34.8–58.6%)	60.0% (48.0–71.1%)	1.16 (0.80–1.69)	0.89 (0.67–1.18)
SA (*n* = 92)	29.3% (16.1–45.5%)	60.8% (46.1–74.2%)	0.75 (0.42–1.34)	1.16 (0.87–1.56)
AC (*n* = 100)	54.2% (39.2–68.6%)	55.8% (41.3–69.5%)	1.22 (0.82–1.83)	0.82 (0.56–1.22)
d) ABPM v Home-monitoring for 1 week (thresholds: ABPM 135/85 mmHg, Home 135/85 mmHg)				
All sample (*n* = 340)	84.0% (77.4–89.2%)	62.4% (54.8–69.5%)	2.23 (1.82 –2.73)	0.26 (0.18–0.37)
WB (*n* = 148)	80.8% (69.9–89.1%)	65.3% (53.5–76.0%)	2.33 (1.68 –3.24)	0.29 (0.18–0.48)
SA (*n* = 92)	85.4% (70.8–94.4%)	54.9% (40.3–68.9%)	1.89 (1.36–2.63)	0.27 (0.12–0.58)
AC (*n* = 100)	87.5% (74.8–95.3%)	65.4% (50.9–78.0%)	2.53 (1.71–3.73)	0.19 (0.09–0.41)

## Discussion

The key finding from this work is that using ABPM as a reference standard, the accuracy of clinic and home measured BP in terms of both diagnostic test performance or ability to confirm controlled BP, does not vary by ethnic group. Standard clinic measurement on three occasions was specific but not sensitive and this did not change materially with different combinations of clinic measurement. Home readings were more sensitive with a modest reduction in specificity but were not good enough for a life-long diagnosis. For people with established hypertension, only home monitoring had reasonable sensitivity with standard (mean clinic23) specific enough – just short of 90% - for daily practice.

This leads us to conclude that for people without hypertension, ABPM remains the choice for diagnosing hypertension compared to the other modes of BP measurement because no other method has sufficient combined sensitivity and specificity on which to base lifelong treatment. Given that systematically measured repeated clinic BP had high specificity, it may be reasonable to treat people with raised mean clinic BP on multiple occasions, but clinic BP could not adequately rule out hypertension and casually measured BP performed poorly. Home-monitored BP was reasonably sensitive and specific but probably not enough so to provide an adequate replacement for ABPM. Sensitivity analyses using BP thresholds of 135/85 mmHg and 140/90 mmHg were undertaken and showed no difference between the ethnic groups.

For people with established hypertension, a sensitivity of 84% from home monitoring is high enough to accept evidence of good control but specificity was low enough to reduce confidence about reacting to high readings.

### Strengths and limitations

This is the first and largest study reporting four modes of BP measurement compared to ambulatory BP monitoring in three different ethnic groups. Participants were both hypertensive and not known to be hypertensive and importantly were not recruited on the basis of previously raised BP which has confounded much of the work on out of office measurement in the past. The attempt to recruit a White Irish group – known to have increased cardiovascular risk – failed due to a lack of numbers of people self-defining as White Irish, despite choosing areas previously identified by census ethnicity responses. This may reflect changes in population since 2001 (the study recruited before 2011 data were available). In light of the results in the groups considered, it seems unlikely that WI would be markedly different. Similarly, people with hypertension responded in greater numbers to our invitations to take part resulting in fewer without a previous diagnosis participating. The key results presented – namely a lack of difference between ethnic groups - were low in heterogeneity between the groups and largely consistent across methods and with or without a diagnosis so appear robust.

The measurement of BP in this study was undertaken in a consistent manner by trained research nurses and facilitators and probably reflects better practice than common outside of specialist centres. Whilst this was a prerequisite for the study, it arguably did not represent usual practice and hence the results might not be generalizable to daily practice. The use of a single reading taken at the first research clinic was designed to both accentuate any white coat effects and also to represent the potential practice of BP measurement in busy clinical settings and proved suboptimal for both diagnosis and ongoing management.

Patients included were chosen to be aged within the range used in NHS Health Checks (40–74) as this is the key age group for whom primary prevention is particularly relevant and for which decisions are commonly made in terms of diagnosis and treatment [17]. Inclusion of older people might have provided information on the diagnosis of hypertension in a group for which evidence for the benefit of treatment is accumulating but would have required different diagnostic thresholds to be applied [18].

The overall performance of clinic (sensitivity 41%/specificity 90%) and home monitoring (73%/76%) for the diagnosis of hypertension are consistent with the receiver operating curves in our previous systematic review which found respectively sensitivities and specificities of clinic (75%/75% (86%/46% for patients around the threshold for diagnosis)) and home (86%/66%) [19].

Since that review, Nasothimiou [20] has found sensitivity and specificity of around 90% for home monitoring in over 600 Greek people referred to a hypertension clinic in comparison to our findings. This may reflect differences in inclusion criteria of that study compared to the community based, diverse population group in the current sample which was not recruited due to their initial BP being raised.

No comparative studies amongst different ethnic groups for the diagnosis of hypertension were apparent from a review of the literature although some studies have grouped together whites with people of Hispanic and/or African origin [21, 22]. The current study therefore represents novel data on which diagnostic decisions taken in a multicultural setting can be based. Given the similarities between the results for those of African Caribbean and South Asian ethnicity with White British it seems unlikely that other ethnicities will have significant differences either, although this would need to be tested.

With greater numbers of individuals in primary care now undergoing out of office BP measurement prior to a diagnosis of hypertension, then this work is reassuring in that it appears appropriate to extend thresholds developed in white populations to South Asian and African Caribbean populations at least.

When making a diagnosis of hypertension in an unselected primary care population, the low sensitivity of clinic measurement means that it cannot reliably be used to rule out hypertension but with high specificity, those found to have high BP on multiple occasions in clinic can be reliably identified and treatment commenced.

In terms of ongoing management of hypertension, the mode of measurement appears more important than the population being measured in terms of variation. In any case, home monitoring would appear to be a reasonable method of ruling out raised BP and in combination with careful clinic measurement provides high specificity in addition. Such a result resonates with the ever growing evidence of improved BP control with the use of home-monitoring, with or without other interventions [23].

In the future, alternative methods of measurement of BP such as central arterial pressure may have increased clinical utility as data accumulate [24]. The feasibility of central pressure measurement in routine clinical practice and its evidence base is increasing.

## Conclusion

Overall the findings confirm those of our previous review [19] that in comparison to ABPM, neither clinic nor home measurement of BP has sufficient clinical value for the diagnosis of hypertension and adds that this is unchanged in South Asian and African Caribbean populations. For ongoing management, combining home and careful clinic measurement appears optimal. Overall, this large study in three different UK ethnic groups has shown that in both the diagnosis and management of hypertension, the method chosen need not be influenced, in terms of test performance at least, in terms of the ethnicity of the individual being tested.

## Additional file

Additional file 1: Diagnostic output of raw data at different BP thresholds. (DOCX 19 kb)

## Abbreviations

ABPM: Ambulatory blood pressure monitoring
AC: Black African Caribbean
BP: blood pressure
Clinic23: Standardised clinic, threshold 140/90 mmHg
Clinic26: Research clinic, threshold 135/85 mmHg
ClinicD1R1: Casual clinic, threshold 140/90 mmHg
CVD: Cardiovascular disease
GP: General practitioner/family medicine
HT: Hypertensive via a clinical code
IMD: Index of multiple deprivation
LR –ve: Negative likelihood ratio
LR + ve: Positive likelihood ratio
NHT: Not known to be hypertensive
SA: South Asians and UK
WB: White British S
WI: White Irish
