# Supplementary material for: Measurement of blood pressure for the diagnosis and management of hypertension in different ethnic groups: one size fits all
Source: BMC Cardiovasc Disord. 2017 Feb 8;17:55. doi: 10.1186/s12872-017-0491-8 (PMC5299651; doi:10.1186/s12872-017-0491-8)
Supplement: Additional file 1: — Diagnostic output of raw data at different BP thresholds. (DOCX 19 kb) [file 12872_2017_491_MOESM1_ESM.docx]

**Additional file 1**

Table S1: Non-Hypertensive: **Diagnostic Output raw data and different thresholds (Complete Data)**

1. **ABPM v Cliniv23** (ABPM 135/85, Clinic23 140/90)

| **Ethnicity** | **Sensitivity (95% CI)** | **Specificity (95% CI)** | **LR +ve (95% CI)** | **LR -ve (95% CI)** |
| --- | --- | --- | --- | --- |
| All sample (n=211) | 39.5% (29.2% - 50.7%) | 90.4% (83.8% - 94.9%) | 4.12 (2.26 - 7.49) | 0.67 (0.56 - 0.80) |
| WB (n=98) | 45.0% (29.3% - 61.5%) | 86.2% (74.6% - 93.9%) | 3.26 (1.57 - 6.76) | 0.64 (0.47 - 0.86) |
| SA (n=55) | 37.5% (15.2% - 64.6%) | 97.4% (86.5% - 99.9%) | 14.63 (1.91 - 111.97) | 0.64 (0.44 - 0.94) |
| AC (n=58) | 33.3% (17.3% - 52.8%) | 89.3% (71.8% - 97.7%) | 3.11 (0.95 - 10.15) | 0.75 (0.56 - 0.99) |

1. **ABPM v Clinic26** (ABPM 135/85, Clinic26 135/85)

| **Ethnicity** | **Sensitivity (95% CI)** | **Specificity (95% CI)** | **LR +ve (95% CI)** | **LR -ve (95% CI)** |
| --- | --- | --- | --- | --- |
| All sample (n=211) | 58.1% (47% - 68.7%) | 83.2% (75.5% - 89.3%) | 3.46 (2.25 - 5.32) | 0.50 (0.39 - 0.65) |
| WB (n=98) | 65.0% (48.3% - 79.4%) | 79.3% (66.6% - 88.8%) | 3.14 (1.81 - 5.46) | 0.44 (0.28 - 0.69) |
| SA (n=55) | 56.3% (29.9% - 80.2%) | 94.9% (82.7% - 99.4%) | 10.97 (2.66 - 45.26) | 0.46 (0.26 - 0.81) |
| AC (n=58) | 50.0% (31.3% - 68.7%) | 75.0% (55.1% - 89.3%) | 2.00 (0.96 - 4.17) | 0.67 (0.44 - 1.01) |

1. **ABPM v ClinicD1R1** (ABPM 135/85, ClinicD1R1 140/90)

| **Ethnicity** | **Sensitivity (95% CI)** | **Specificity (95% CI)** | **LR +ve (95% CI)** | **LR -ve (95% CI)** |
| --- | --- | --- | --- | --- |
| All sample (n=211) | 65.1% (54.1% - 75.1%) | 74.4% (65.8% - 81.8%) | 2.54 (1.82 - 3.56) | 0.47 (0.35 - 0.64) |
| WB (n=98) | 72.5% (56.1% - 85.4%) | 72.4% (59.1% - 83.3%) | 2.63 (1.66 - 4.16) | 0.38 (0.22 - 0.64) |
| SA (n=55) | 75.0% (47.6% - 92.7%) | 84.6% (69.5% - 94.1%) | 4.88 (2.22 - 10.73) | 0.30 (0.13 - 0.70) |
| AC (n=58) | 50.0% (31.3% - 68.7%) | 64.3% (44.1% - 81.4%) | 1.40 (0.76 - 2.58) | 0.78 (0.49 - 1.22) |

1. **ABPM v** home (ABPM 135/85, home 135/85)

| **Ethnicity** | **Sensitivity (95% CI)** | **Specificity (95% CI)** | **LR +ve (95% CI)** | **LR -ve (95% CI)** |
| --- | --- | --- | --- | --- |
| All sample (n=211) | 72.1% (61.4% - 81.2%) | 76.0% (67.5% - 83.2%) | 3.00 (2.14 - 4.21) | 0.37 (0.26 - 0.52) |
| WB (n=98) | 67.5% (50.9% - 81.4%) | 79.3% (66.6% - 88.8%) | 3.26 (1.89 - 5.64) | 0.41 (0.26 - 0.65) |
| SA (n=55) | 87.5% (61.7% - 98.4%) | 79.5% (63.5% - 90.7%) | 4.27 (2.24 - 8.13) | 0.16 (0.04 - 0.58) |
| AC (n=58) | 70.0% (50.6% - 85.3%) | 64.3% (44.1% - 81.4%) | 1.96 (1.13 - 3.40) | 0.47 (0.25 - 0.86) |

# Table S2: Hypertensive: Diagnostic Output raw data and different thresholds (Complete Data)

1. **ABPM v Cliniv23** (ABPM 135/85, Clinic23 140/90)

| **Ethnicity** | **Sensitivity (95% CI)** | **Specificity (95% CI)** | **LR +ve (95% CI)** | **LR -ve (95% CI)** |
| --- | --- | --- | --- | --- |
| All sample (n=340) | 41.1% (33.5% - 49.1%) | 88.1% (82.4% - 92.5%) | 3.46 (2.23 - 5.39) | 0.67 (0.58 - 0.77) |
| WB (n=148) | 39.2% (28.0% - 51.2%) | 89.2% (79.8% - 95.2%) | 3.63 (1.78 - 7.40) | 0.68 (0.56 - 0.83) |
| SA (n=92) | 34.1% (20.1% - 50.6%) | 84.3% (71.4% - 93.0%) | 2.18 (1.01 - 4.68) | 0.78 (0.61 – 1.00) |
| AC (n=100) | 50.0% (35.2% - 64.8%) | 90.4% (79.0% - 96.8%) | 5.20 (2.16 - 12.54) | 0.55 (0.41 - 0.74) |

1. **ABPM v Clinic26** (ABPM 135/85, Clinic26 135/85)

| **Ethnicity** | **Sensitivity (95% CI)** | **Specificity (95% CI)** | **LR +ve (95% CI)** | **LR -ve (95% CI)** |
| --- | --- | --- | --- | --- |
| All sample (n=340) | 60.7% (52.8% - 68.3%) | 78.0% (71.1% - 83.8%) | 2.76 (2.04 - 3.73) | 0.50 (0.41 - 0.62) |
| WB (n=148) | 56.8% (44.7% - 68.2%) | 71.6% (59.9% - 81.5%) | 2.00 (1.32 - 3.02) | 0.60 (0.45 - 0.81) |
| SA (n=92) | 58.5% (42.1% - 73.7%) | 78.4% (64.7% - 88.7%) | 2.71 (1.51 - 4.86) | 0.53 (0.36 - 0.78) |
| AC (n=100) | 68.8% (53.7% - 81.3%) | 86.5% (74.2% - 94.4%) | 5.11 (2.5 - 10.44) | 0.36 (0.23 - 0.56) |

1. **ABPM v ClinicD1R1** (ABPM 135/85, ClinicD1R1 140/90)

| **Ethnicity** | **Sensitivity (95% CI)** | **Specificity (95% CI)** | **LR +ve (95% CI)** | **LR -ve (95% CI)** |
| --- | --- | --- | --- | --- |
| All sample (n=340) | 68.1% (60.4% - 75.2%) | 58.2% (50.6% - 65.5%) | 1.63 (1.33 - 2.00) | 0.55 (0.42 - 0.71) |
| WB (n=148) | 71.6% (59.9% - 81.5%) | 60.8% (48.8% - 72.0%) | 1.83 (1.33 - 2.51) | 0.47 (0.31 - 0.70) |
| SA (n=92) | 53.7% (37.4% - 69.3%) | 52.9% (38.5% - 67.1%) | 1.14 (0.76 - 1.71) | 0.88 (0.58 - 1.33) |
| AC (n=100) | 75.0% (60.4% - 86.4%) | 59.6% (45.1% - 73.0%) | 1.86 (1.28 - 2.68) | 0.42 (0.24 - 0.72) |

1. **ABPM v** home (ABPM 135/85, home 135/85)

| **Ethnicity** | **Sensitivity (95% CI)** | **Specificity (95% CI)** | **LR +ve (95% CI)** | **LR -ve (95% CI)** |
| --- | --- | --- | --- | --- |
| All sample (n=340) | 84.0% (77.5% - 89.3%) | 63.3% (55.7% - 70.4%) | 2.29 (1.87 - 2.81) | 0.25 (0.17 - 0.36) |
| WB (n=148) | 81.1% (70.3% - 89.3%) | 66.2% (54.3% - 76.8%) | 2.40 (1.71 - 3.36) | 0.29 (0.17 - 0.47) |
| SA (n=92) | 85.4% (70.8% - 94.4%) | 54.9% (40.3% - 68.9%) | 1.89 (1.36 - 2.63) | 0.27 (0.12 - 0.58) |
| AC (n=100) | 87.5% (74.8% - 95.3%) | 67.3% (52.9% - 79.7%) | 2.68 (1.79 - 4.01) | 0.19 (0.09 - 0.40) |
